# Buttock tissue response to loading in men with spinal cord injury

**DOI:** 10.1371/journal.pone.0191868

**Published:** 2018-02-07

**Authors:** Sharon Eve Sonenblum, Stephen H. Sprigle

**Affiliations:** 1 George W. Woodruff School of Mechanical Engineering, Georgia Institute of Technology, Atlanta, Georgia, United States of America; 2 School of Industrial Design, Georgia Institute of Technology, Atlanta, Georgia, United States of America; University of Illinois at Urbana-Champaign, UNITED STATES

## Abstract

**Objective/Background:**

Despite the fact that most people with a spinal cord injury who use a wheelchair for mobility are considered at-risk for pressure ulcer (PrU) development, there still exists a spectrum of risk amongst this group. Efforts to differentiate risk level would benefit from clinical tools that can measure or predict the buttocks response to loading. Therefore, the goal of this study was to identify how tissue compliance and blood flow were impacted by clinically-measurable risk factors in young men with SCI.

**Methods:**

Blood flow at the ischial tuberosity was measured using laser Doppler flowmetry while the seated buttock was unloaded, and loaded at lower (40–60 mmHg) and high (>200 mmHg) loads. Tissue compliance of the buttock was measured using the Myotonometer while subject were lifted in a Guldmann Net.

**Results:**

Across 28 participants, blood flow was significantly reduced at high loads, while no consistent, significant changes were found at lower loads. At 40–60 mmHg, blood flow decreased in participants with a pressure ulcer history and lower BMI, but stayed the same or increased in most other participants. The buttock displaced an average of 9.3 mm (2.7 mm) at 4.2 N, which represented 82% (7%) of maximum displacement. BMI was related to the amount of buttock tissue displacement while smoking status explained some of the variation in the percent of max displacement.

**Conclusion:**

Wide variability in tissue compliance and blood flow responses across a relatively homogeneous population indicate that differences in biomechanical risk may provide an explanation for the spectrum of PrU risk among persons with SCI.

## Introduction

Pressure ulcers (PrUs) are a leading secondary complication of spinal cord injury (SCI), affecting more than 50% of people with SCI at some point in their lives. [1] The costs of PrUs extend far beyond the medical costs incurred for treatment. Personal and societal costs from inactivity, as well as missed educational, vocational, and recreational pursuits are equally important.

External pressure has long been identified as defining cause of PrUs. [2, 3] The mechanisms, however, by which external pressure deforms the internal soft tissue and consequently leads to tissue breakdown have not been established, [4, 5] but are believed to include cell damage directly from prolonged deformation and deformation-induced ischaemia of soft tissues. [6–8]

While sitting is likely to induce deformation and ischaemia for all people, the specific tissue and blood flow responses to loading vary across individuals. For example, studies have identified differences in tissue stiffness according to body mass index (BMI) and diagnosis. [9–11] Computational models have been used to predict the influence of individual characteristics such as body mass index, tissue stiffness and thickness on the amount of internal stress and strain that results from sitting. [12–15] [16–22] Furthermore, researchers have found significant variation in the amount of applied pressure required to occlude blood flow. [23–26] It is believed that this variation is likely to depend on individual characteristics that contribute to the tissue’s response to loading and influence a person’s biomechanical risk. [5, 27–29]

Biomechanical Risk can be defined as the intrinsic characteristic of an individual’s soft tissues to deform in response to extrinsic applied forces. Biomechanical risk results from changes in tissue type, tissue mass and/or tissue stiffness. An increase in biomechanical risk is embodied by greater tissue deformations, which may place the individual at a greater risk of tissue breakdown. [8, 16, 30] The variation in biomechanical risk is a likely explanation for why there exists a spectrum of PrU risk within a high-risk diagnostic group (e.g., spinal cord injury). Over the years, dozens of clinical risk factors have been identified, many of which may impact biomechanical risk. For example, established risk factors such as age, [31–33] atrophy, low body mass index, [34, 35] spasticity, [32] and body weight [36] have direct relationships to the bulk mechanical properties of tissues. Other identified risk factors may also impact aspects of tissue mechanics by changing the mechanical or structural characteristics of the skin and underlying tissue. These include nutrition, [32, 37, 38] edema, infection/fever, [33, 35] smoking, [37, 39] hypoalbuminemia, [34, 35] lymphopenia, [34, 35] fever, [34, 35] and continence, [31, 38] to name a few.

Risk factors gleaned from medical charts have been assembled into PrU risk assessment tools (e.g., Braden, Norton) and clinical guidelines [40, 41] that identify clinical presentations that predispose people to pressure ulcers. But they do not consider the tissue’s response to load, the defining characteristic of PrUs. As such, risk assessment tools inform the clinician which patients should be watched more closely but are not designed to inform clinicians about personalized interventions. [42] Clinical guidelines extend beyond risk assessment and identify techniques and interventions that should be considered when developing preventative treatment plans. At this time, research has yet to directly link clinical risk factors to their role in PrU etiology, thereby hindering a personalized prevention approach. Information regarding how risk factors affect tissue’s response to load, and, by extension, a person’s tolerance to load, will empower clinicians to make more informed decisions. By knowing if a client is at high or low biomechanical risk, clinicians can better select cushions and define pressure relief interventions. [40]

Therefore, this study seeks to extend our understanding of PrU risk in individuals with SCI in order to influence clinical practice. The goal of this study was to identify clinically-measurable characteristics that can be used to predict the buttocks tissue response to loading. Specifically, we sought to identify how tissue compliance and blood flow were impacted by clinically-measurable risk factors in men with SCI.

## Methods

### Participants

A group of functionally similar participants at high risk for pressure ulcers was recruited to reduce the variability across age, gender and diagnosis, as a means to focus on other risk factors that might contribute to blood flow and tissue responses. Specifically, we recruited a convenience sample of 35 participants who met the following inclusion criteria: men ages 18–40, a diagnosis of SCI, more than 2 years post injury, used a wheelchair as their primary means of mobility, and had no open pressure ulcers. IRB approval for this study was received from the Georgia Institute of Technology and Shepherd Center, and participants provided written consent prior to beginning their involvement in the study.

### Test environment

**Fig 1:**
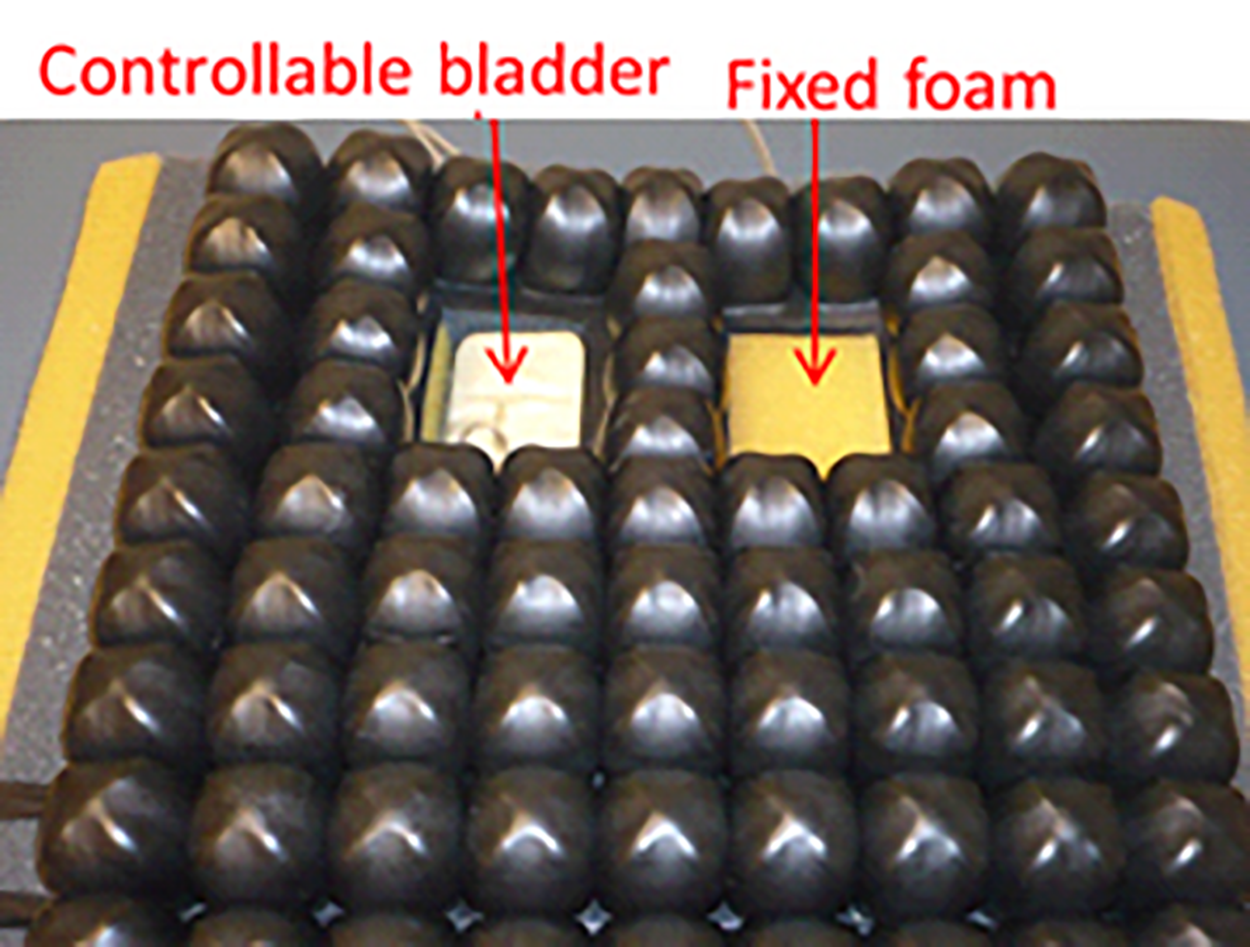
Custom cushion for testing blood flow response to loading.

To investigate the superficial blood flow response to loading and unloading, a custom test environment was developed that allowed us to load and unload the ischial tuberosity (IT) region of participants’ buttock while they sat in an upright posture. A custom wheelchair cushion was created, which contains a bladder under one IT region of a seated participant (Fig 1). Inflating and deflating the bladder provided full control over the pressure at the IT. Loading was controlled at the right IT, unless there was a history of pressure ulcers at that location, in which case the bladders were swapped and the left IT was studied. Target interface pressures were identified using measurements from a small (3”x5”) Tekscan (Tekscan, Inc. South Boston, MA) sensor adhered to the bladder. The superficial blood flow was measured by the PeriFlux 5010 Laser Doppler Perfusion Monitor (Perimed AB, Sweden) with a custom probe (12.5 (l) x9.5 (w) x 2.3mm (t)) attached to the buttock [43, 44] using a medical grade double-sided adhesive. The custom cushion was placed on top of an adjustable wheelchair frame that could be configured for the participant, with a 90° knee angle, 100° seat to back angle and 5° of seat tilt.

### Test protocol

**Fig 2:**
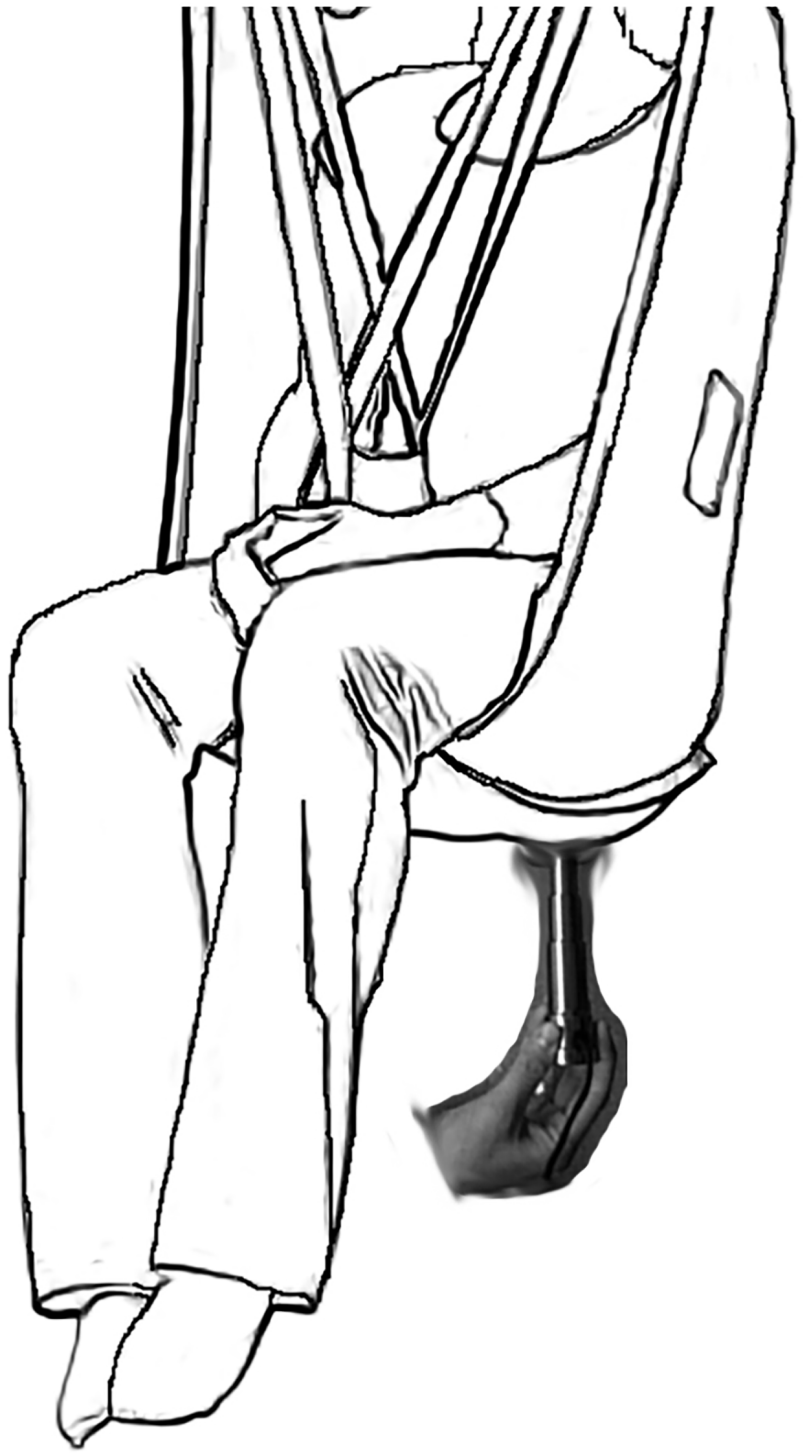
Myotonometer in use while subject is lifted in the Guldmann net.

The test protocol was approved by the local Institutional Review Boards and informed consent was received from all participants prior to beginning the study. Immediately following consent, the research therapist took the participants’ blood pressure. Then participants, wearing a pair of loosely fitted boxer shorts, were lifted in a Guldmann net to provide access to the ischial region (Fig 2). The Guldmann net was set up to maintain a relatively upright, seated posture. With the subject lifted using a Guldmann ceiling mounted hoist system, the apex of the IT was palpated and the laser Doppler probe was attached at the apex. Subjects were lowered back onto the test cushion and the configuration of the wheelchair was checked and adjusted as needed. The location of the laser Doppler probe was palpated to confirm its position beneath the IT and on top of the controllable bladder. The net was left in place and participants were asked not to move for the duration of the study.

**Fig 3:**
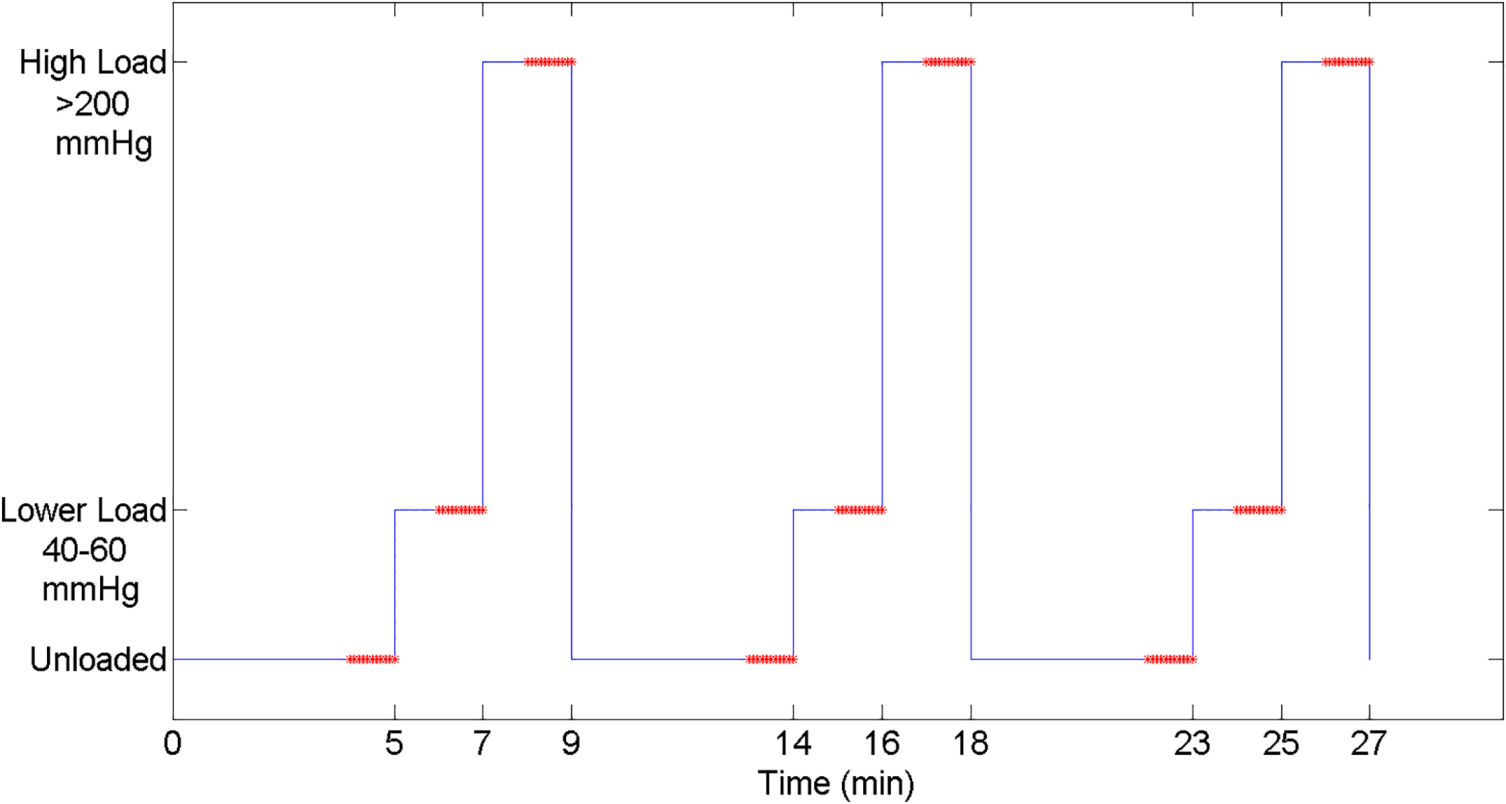
Loading pattern used for measuring blood flow. Red asterisks indicate the 60 seconds of analyzed blood flow in each loading condition.

The data collection protocol involved three trials of sequences that began with the IT unloaded for 5 minutes, followed by 2 minutes at a lower load (40–60 mmHg) and 2 minutes at a high load (> 200 mmHg, Fig 3). 2 minutes of loading was used to minimize the development and duration of any hyperaemic response following loading, while optimally permitting blood flow to reach steady state. We anticipated that 4 minutes of loading followed by 5 minutes unloaded would provide ample time to reach steady state between trials. This assumption was evaluated during analysis. The Laser Doppler Flowmeter was configured to record the blood flow flux at a sampling rate of 32 Hz and the interface pressure sensor measured the interface pressure at 1 Hz. Flux is a unitless value that represents both the concentration of moving blood cells and the average velocity of these cells in a region of tissue approximately 1mm^3^. It is typically reported in arbitrary units (AU).

After collection of blood flow data, subjects were lifted in the Guldmann net and the sensors were removed. Next tissue compliance was collected using the Myotonometer. The Myotonometer was manually pushed vertically on the buttock towards the IT. The tissue was loaded to 14.7 N (1.5 kgf) and unloaded for 8 continuous cycles. This test was repeated 2 more times, with a rest period of 1 minute between tests.

Participants also filled out a survey addressing characteristics hypothesized as having a relationship to blood flow response (i.e., BMI, level of injury, and smoking status) as well as other important characteristics and demographics. Finally, the hospital’s phlebotomist drew blood, and serology results were obtained from the lab to evaluate total lymphocyte count, hematocrit, and serum albumin.

### Independent variables

Decades of pressure ulcer risk literature have identified hundreds of intrinsic risk factors. [45] For this study, we identified 7 known pressure ulcer risk factors that we believed might impact tissue compliance or blood flow response to loading. These included level of injury, [31, 32, 38] body mass index (BMI), [34] blood pressure, smoking status, [1, 31, 37, 46] hematocrit, [47] serum albumin, [31, 47] and lymphopenia (< 1500 cells/mL). [34] We also investigated race, time since injury, and the history of a pelvic pressure ulcer, defined as any stage pressure ulcer at the ischium, sacral-coccyx region, or trochanter, as self-reported by the participant.

### Dependent variables—Tissue compliance and blood flow

Dependent variables were grouped into measures of tissue compliance and blood flow, where tissue compliance was also considered as an independent variable to predict blood flow. The two areas are described separately.

#### Tissue compliance

The Myotonometer outputs the tissue displacement at fixed loads, allowing for the calculation of a number of different metrics. We chose to study two orthogonal metrics of tissue compliance.

1. ButtockDisplacement: the displacement of the buttock tissue at the apex of the IT with the application of 4.2 N of load (which given the contact area of device, equates to a little over 200 mmHg of interface pressure); and
2. %MaxDisplacement: reflects the ratio (in percent) of displacement at 4.2 N to displacement at 14.7 N. This is a measure of how “bottomed out” the tissue is at 4.2 N.

#### Blood flow

Blood flow signals were filtered with a 2^nd^ order, low pass Butterworth filter with a cutoff frequency of 1 Hz. [44] Blood flow was further analyzed by taking the average flow from the final 60 seconds in the low and high loaded conditions and dividing by the preceding unloaded blood flow to produce a normalized blood flow. Normalized blood flow is important because Laser Doppler measures blood flow in arbitrary units, making comparisons of absolute measures across subjects more difficult than relative changes.

### Data analysis

All variables were evaluated for normality using a Kolmogorov-Smirnov test, with a p-value < 0.05 needed to reject the null hypothesis of normality. The normal distribution was a good fit to all variables, so parametric analyses were used as follows.

The slope of the blood flow was calculated over the final 60 seconds as an indication of whether or not flow had reached steady state. A slope of 0 would indicate steady state, while a slope < 0 would suggest the blood flow was still dropping from the hyperaemic response towards a steady state baseline. The median normalized blood flow across 3 trials during both loading conditions was run through a one-sample t-test (hypothesis mean = 1) to determine if there was a change in blood flow in response to loading, with p<0.05 considered significant.

To determine which risk factors impacted the tissue compliance, two step-wise regression models were run with 5 risk factors (i.e., level of injury, BMI, mean arterial pressure, smoking status, and lymphopoenia status) as the inputs and the median ButtockDisplacement and %MaxDisplacement (across 3 trials) as the outputs. Participants’ level of injury was categorized into cervical or non-cervical (thoracic or lumbar) injuries. P<0.05 was selected for inclusion of a variable. The remaining two variables (hematocrit and serum albumin) were not included in the modeling because almost all subjects had normal levels, and the variability across subjects was very small.

A similar approach was used to evaluate blood flow, although inputs included the 5 risk factors and the two measures of buttock tissue compliance. Outputs were the median normalized blood flow (across 3 trials) at the lower and high pressure conditions.

Secondary analysis of the relationships between additional risk factors (e.g., pressure ulcer history, race) and tissue compliance and blood flow were investigated independently using a one-way ANOVA, while correlations between time since injury and tissue compliance and blood flow were also investigated.

## Results

### Subject population

Complete data was collected on 34 subjects. One participant did not complete the study because bruising was identified on his ischium prior to data collection. Another 6 subjects were removed from analysis because they ambulated within their homes daily despite using a wheelchair for most of their mobility, resulting in analysis of 28 full time wheelchair users. As per the inclusion criteria, all participants were male ages 18–40 more than 2 years post SCI (Table 1). The hematocrit and albumin levels of participants had little variability, with only one subject being anemic, and none having hypoalbuminemia.

**Table 1:** Subject characteristics.

Participant Characteristics (n = 28)	
**Characteristic**	**Number (%)**
Race	
Black/African American	17 (61)
White	9 (32)
Hispanic or Latino	2 (7)
Visible Blanching	24 (86)
Current Smokers (n (%))	9 (32)
Lymphopenia (n (%))	8 (29)
Cervical Injury (n (%))	13 (46)
Incomplete Injury	10 (36)
Spasticity	24 (86)
Any Controlled Movement Below the Waist	8 (29)
Presence of Sensation at the Buttocks	10 (36)
History of Pelvic Pressure Ulcer	17 (61)
**Characteristic**	**Mean (SD)**
BMI (kg/m^2^)	23.5 (5.1)
Years Post Injury	10.5 (5.0)
Mean Arterial Pressure (mmHg)	86.8 (16.0)
Hemoglobin (g/dL)	13.9 (1.1)
Serum Albumin (g/DL)	4.0 (0.3)

### Tissue compliance

**Fig 4:**
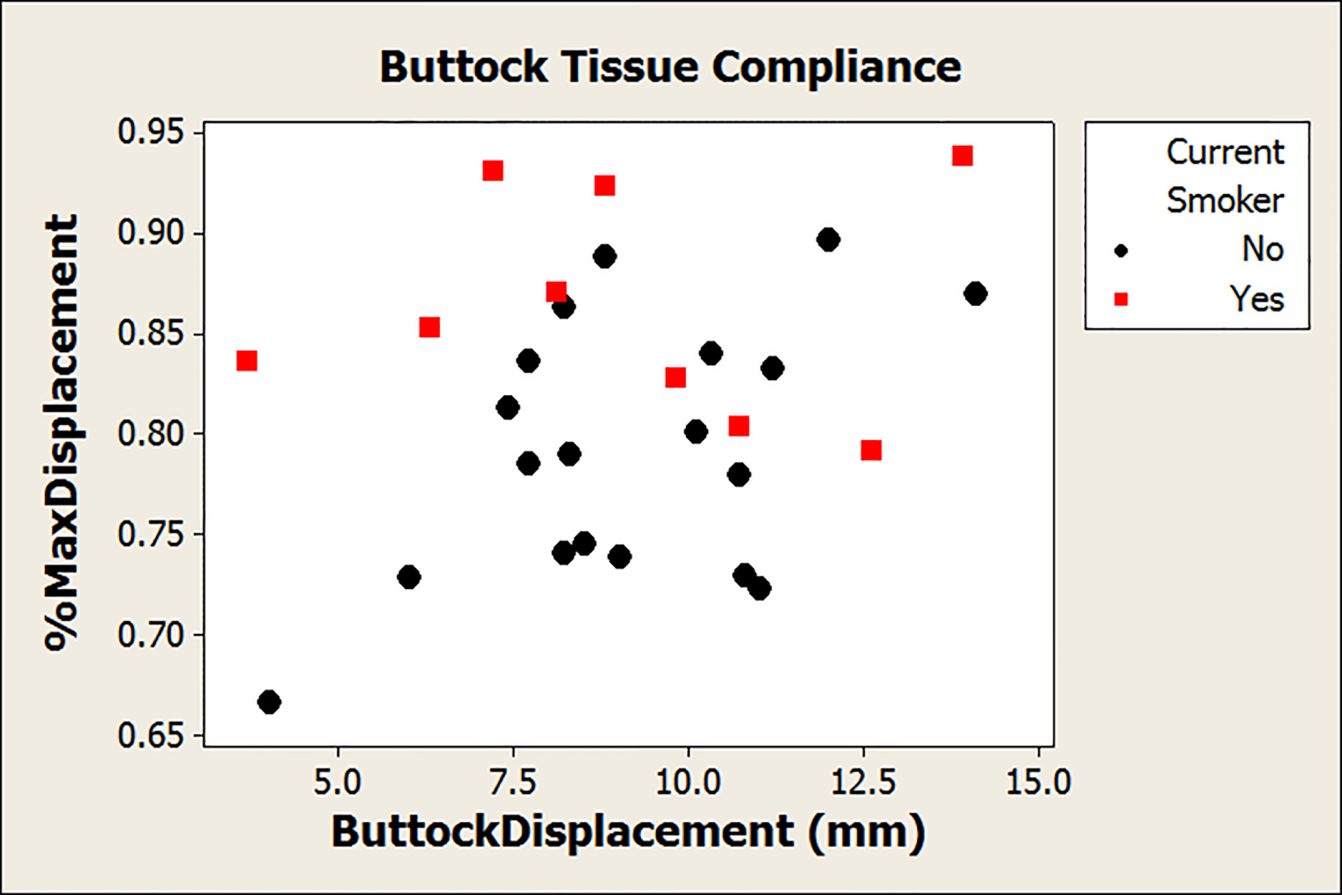
Buttock tissue compliance across subjects.

Tissue compliance varied widely across 35 participants (Table 2, Fig 4). In the regression model for ButtockDisplacement, only BMI was related to the amount of buttock displacement (β = 0.299, 95% CI [0.106, 0.492]). In other words, a clinically important change in BMI of 10% of the mean (2.4 kg/m^2^) corresponds to an additional 0.6 mm of displacement, an increase of 7%. The model for %MaxDisplacement also included only one risk factor—smoking status (β = 0.070, 95% CI [0.018, 0.122]). Current smokers demonstrate greater tissue compliance, experiencing 86 ± 5% of maximum displacement at 4.2N, compared with only 79 ± 6% for non-smokers (Fig 4).

**Table 2:** Tissue compliance varied widely across subjects.

Tissue Compliance Metric	Mean (SD)	Range
ButtockDisplacement (mm)	9.3 (2.7)	3.7–14.6
%MaxDisplacement (%)	82 (7)	67–94

There was no difference in tissue compliance, neither ButtockDisplacement nor %MaxDisplacement, according to pressure ulcer history (p = 0.955 and p = 0.942, respectively). Similarly, tissue compliance was not correlated with time since injury. There was a small, but significant difference between the %MaxDisplacement of participants who were white (mean (SD) = 85 (5)) and those who were not (mean (SD) = 80 (7), p = 0.041).

### Blood flow

As described in the methods, average flow was calculated over the final 60 seconds of each loading condition. To assess whether this timeframe reflected steady-state blood flow, the slope of the blood flow data was compared to zero for each trial (Table 3). In all loading conditions, the slope was not significantly different than zero.

**Table 3:** Blood flow slope during the final 60 seconds at each condition was calculated to determine if steady state was reached.

Loading Condition	Blood Flow Slope (AU/min) Mean(SD)	95% Confidence Interval
Unloaded	-0.39 (2.59)	(-0.95, 0.17)
Low	-0.21 (2.09)	(-0.67, 0.24)
High	0.17 (1.00)	(-0.05, 0.39)

When averaged across participants, blood flow did not experience a significant change from unloaded with a lower load was applied to the buttock (Table 4). At high loads, however, blood flow was significantly reduced.

**Table 4:** Normalized blood flow at high and lower loads.

Loading Condition	Blood Flow mean(SD)	95% Confidence Interval
Low	1.1 (0.6)	(0.8, 1.3)
High	0.3 (0.3)	(0.2, 0.4)

**Fig 5:**
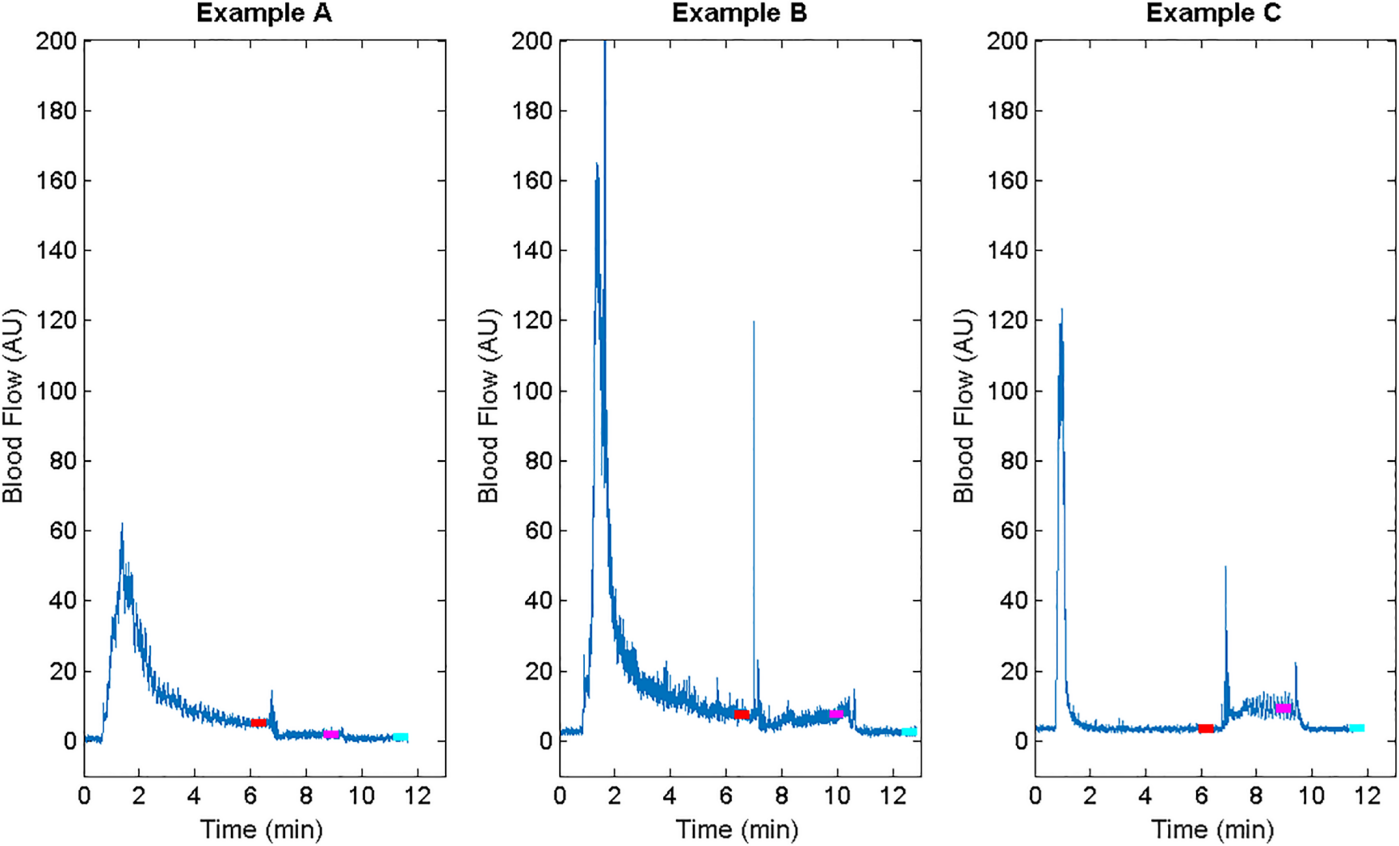
Three “typical” blood flow responses seen across subjects include a decrease in blood flow at lower load compared with higher load (Example A), similar blood flow at lower load (Example B), and an increase in blood flow with lower load (Example C). AU = Arbitrary Units.

Fig 5 demonstrates 3 typical blood flow responses (observed clearly in at least 5 participants each). In each response, unloading the buttock was met with a large hyperaemic response, followed by a decay towards steady state blood flow. In nearly all participants, blood flow decreased when a high load was applied. In Example A, the presentation is notable due to the slowly decaying hyperaemic response and a decrease in blood flow at the lower load compared with unloaded. Example B demonstrates a somewhat faster hyperaemic response and no change in blood flow when a lower load was induced. Example C, on the other hand, illustrates a hyperaemic response that returns quickly to steady state, followed by an increase in blood flow with the addition of a lower load.

Regression modeling to predict blood flow responses was made difficult by the high inter-subject variability and the differences in types of responses noted above (Table 4 and Fig 5). None of the risk factors investigated were found to be significant in the models for blood flow at high load. Only one of the risk factors identified a priori had a statistically significant role in the regression models at lower load—BMI (β = 0.047, 95% CI [0.005, 0.090]). However, the contributions of BMI to blood flow were small, in that a change in BMI of 10% of the mean (2.4 kg/m^2^) was associated with an increase in normalized blood flow of 0.1 AU at lower loads.

Blood flow at lower loads differed according to whether or not participants had a history of pelvic pressure ulcers. Participants with no history of pressure ulcers had greater blood flow (mean (SD) = 1.5 (0.7)) at lower loads compared with participants with a history of pressure ulcers (mean (SD) = 0.8 (0.4), p = 0.006, 95% CI for difference = [0.2, 1.2].) Because some participants experienced an increase in blood flow (normalized flow > 1) while others experienced a decrease in blood flow (normalized flow < 1) at lower loads, we grouped the data according to the direction of response. The odds of experiencing a decrease in blood flow were significantly greater if the participant had experienced a pressure ulcer previously (odds ratio = 14.62, p = 0.006, 95% CI = [2.19, 97.61]). Blood flow at lower and high loads were not related to time since injury nor did it differ according to race.

## Discussion

Few studies of pressure ulcer risk seek to understand risk based on the buttocks response to external loading, which is the defining characteristic of a pressure ulcer. Instead, most focus on chart reviews and factors that only indirectly influence pressure ulcer development. This study selected a relatively homogeneous population—younger men with chronic SCI—in order to study buttocks tissue response to loading and extend the state of the knowledge about risk using the tissue response to load. The study found multiple risk factors that contributed to buttock tissue response to loading, and found considerable variation in the buttock response across individuals. Clinically, this implies that persons with SCI have different levels of pressure ulcer risk despite similarity of gender and age. Therefore, they may have very different needs in terms of cushion prescription and pressure relief interventions. In other words, their interventions may be personalized according to measurements of their tissue response to loading.

The precise mechanisms by which internal loading and physiological responses lead to pressure ulcers are not known. However, current evidence suggests that damage can result directly from the deformation, [48, 49] or the result of impairment to blood flow that results from deforming tissue under load. [50, 51] Therefore, tissue that is more prone to deformation during sitting is likely to be at greater risk for PrU development. This study found that ButtockDisplacement, a measure of tissue compliance, and normalized blood flow at lower loads, varied with BMI. Individuals with higher BMI experienced a greater magnitude of deformation of the bulk tissue at the IT and slightly increased blood flow at lower loads. While these results support the existence of differences in tissue response according to BMI, how those changes relate to pressure ulcer risk requires further investigation, particularly as the changes to tissue deformation may increase risk while changes to blood flow may not. Furthermore, future work would benefit from including a larger population of underweight individuals.

Another interesting observation was that %MaxDisplacement, which is related to the load at which the tissue reaches maximum deformation, was impacted by smoking and race. Being a smoker resulted in the tissue bottoming out at a lower load than non-smokers, meaning there was less cushioning left in the tissue to react when greater loads were experienced, such as during a transfer when larger impact forces are experienced. The loss of this safety factor could result from degradation of elastic fibres in the tissue, [52] and suggests that the mechanism by which smoking impacts PrU development extends beyond its impact on blood flow responses. [46] A similar difference was seen between participants who were white and those who were Hispanic or African-American, with white participants bottoming out at a slightly lower load than other participants.

Superficial blood flow responses to loading demonstrated even greater variability across subjects, making statistical modeling more difficult. At high loads, blood flow was significantly reduced for all subjects, with an average reduction of 70%, suggesting that everyone could be at risk of tissue breakdown at such loads. Therefore, it may not be valuable to further investigate differences in responses at high loads. Of greater importance is studying the varied response to loading at lower loads.

This study applied 40–60 mmHg to investigate tissue’s response to a lower load and found that it was much more likely for blood flow to decrease with load in participants with a pressure ulcer history than those without. The nature of this cross-sectional study makes it impossible to assess whether a reduced blood flow response was present at the time of pressure ulcer development. Nevertheless, this study demonstrated that while everyone appears to be at risk of tissue breakdown at very high loads, the blood flow response at clinical loads between 40 and 60 mmHg may be an indicator of increased risk that can be used to personalize prevention strategies. In 1990, Bader described a similar impaired response in blood oxygenation in persons with disabilities compared with able-bodied individuals. [53]

There are additional potential clinical implications to the differences in blood flow observed. Participants such as those presented in Fig 5C, who experienced an increase in blood flow with lower loads, may find that weight shifts that induce partial unloading are sufficient for them, although further investigation into this is warranted. On the other hand, participants who demonstrate a decrease in blood flow at lower loads may require complete unloading. Improving our understanding of the differences in biomechanical risk and our ability to predict that risk across individuals has the potential to inform pressure ulcer prevention strategies.

### Limitations

Blood flow measurements themselves represent a limitation, as Laser Doppler measurements only include superficial blood flow and do not describe perfusion of deeper tissues near the ischial tuberosities. However, the finding that there is a relationship between blood flow at lower loads and pressure ulcer history or risk suggests that there is some validity to measuring superficial blood flow. Previous pressure ulcers were often experienced at a different location than the IT that was loaded and measured, suggesting that the blood flow response may be indicative of the overall, systemic response to loading.

In this study, we chose to analyze normalized blood flow, because it offers numerous benefits such as the ability to compare the dimensionless metric of blood flow across subjects and trials. However, while normalizing permits analysis of the acute impacts of loading, it masks potential impacts of loading over time. That is, we observed that blood flow in the unloaded and low conditions increased over 3 study trials, but because the increase in flow was proportional, no such increase was seen in the normalized blood flow measurements. A more in depth analysis of the impact of multiple trials of alternating high, low, and no loading was beyond the scope of this study but would be beneficial in the future.

Finally, the population and sample size present a limitation. Expanding on age and sex would allow for greater generalizability of our results. The decision to exclude individuals who were partially ambulatory from analysis also helped to make the population more uniform, but studying that cohort may inform us about any protective biomechanical changes associated with daily walking in wheelchair users. Increasing the sample size would also allow for the investigation of more risk factors as well as an improved understanding of potential relationships between risk factors.

## Conclusions

This study extends the state of knowledge about PrU risk by expanding risk assessment beyond chart reviews to consider the impact of load. Despite the fact that most people with an SCI who use a wheelchair for mobility are considered at-risk for PrU development, there still exists a spectrum of risk amongst this group. This study identified a difference in blood flow response to loading between people with and without a history of PrU, a difference that may serve as an indication of risk or explanation for the differences in risk across individuals with SCI. Tissue compliance and blood flow may be helpful clinical predictors of Biomechanical Risk, but a more complete description of Biomechanical Risk is needed. To that end, further investigation into the deformation of the entire buttocks is ongoing.
